# A long lasting puzzle for −7/7q− syndrome

**DOI:** 10.18632/oncotarget.1763

**Published:** 2014-01-22

**Authors:** Hiroaki Honda, Toshiya Inaba

**Affiliations:** Department of Disease Model, Research Institute for Radiation Biology & Medicine, Hiroshima University; Department of Molecular Oncology and Leukemia Program Project, Research Institute for Radiation Biology & Medicine, Hiroshima University

Chromosomal aberrations are major characteristics of cancer cells. In hematopoietic malignancies, while chromosomal translocations are most frequently observed, chromosomal deletions, such as −5/5q− and −7/7q−, also account for a significant portion of the diseases of myeloid lineage and correlate with a poor prognosis [1].

−7/7q− is frequently observed in patients with radiation- and/or chemotherapy-induced myelodysplastic syndrome (MDS) and its related diseases [1]. Previous efforts identified two commonly deleted regions; one is around band 7q22 and the other around 7q34. To isolate gene(s) responsible for −7/7q− syndrome, we focused on the former region, analyzed it with high microarray comparative genomic hybridization (mCGH) and identified a microdeletion at subband 7q21.3 [2]. This region contains three genes, *sterile α motif*(*SAM*) domain-9 (*Samd9*), *Samd9L*, and *Miki*(*LOC253012*), which are deleted in one allele in ~25% of MDS and AML patients with 7q− [2]. *Samd9*and *Samd9L*are highly homologous in humans (~60%) and mice have only *Samd9L*, strongly suggesting that these genes were evolutionally divided from a common ancestor.

In our recent paper, we generated and analyzed mice deficient in *Samd9L*and found that both heterozygous (*Samd9L*^+/−^) and homozygous (*Samd9L*^-/-^) mice developed myeloid malignancies closely resembling to human diseases with −7/7q− after a long latent period (>12 months) [3]. They exhibited abnormal white blood cell counts (mainly leukocytopenia and rarely leukocytosis), anemia and occasionally low platelet number. The peripheral blood smear showed myelodysplasia, including Pseudo-Pelger-Huet anomaly, Howel-Jolly body, and giant platelets. The spleen was frequently enlarged with proliferation of mature and immature myeloid cells, and the bone marrow was normo~hypercellular with maturation arrest in myeloid and/or erythroid lineage(s). These pictures show cardinal features of MDS and related diseases, allowing us to consider the mice as a model for human −7/7q− syndrome [3].

It is of note that both *Samd9L*^+/−^ and *Samd9L*^−/−^ mice developed MDS and related diseases, and the hematopoietic tissues of diseased *Samd9L*^+/−^ mice still possessed functional *Samd9L*allele (namely, expressed SAMD9L protein with no mutation) [3]. In addition, *Samd9L*^+/−^ hematopoietic cells, as well as *Samd9L*^−/−^ cells, exhibited prolonged survival in the colony-replating assay and enhanced reconstitution activity in the competitive repolutating assay [3]. These results indicate that *Samd9L*is a haploinsufficient gene and reduced expression of SAMD9L is responsible for the disease developed in the mice.

Since SAMD9L-deficient hematopoietic cells possessed an enhanced sensitivity to cytokines, we investigated SAMD9L function in cytokine-mediated intracellular signaling. SAMD9L was shown to bind to RGL2, an endosomal protein, which prompted us to examine whether SAMD9L is involved in the endocytosis, degradation, and recycling of cytokine receptors. Kinetic and functional analyses of SAMD9L revealed that it traces endocytosed cytokine receptors and promotes receptor degradation through facilitating homotypic fusion with endosomes [3]. Haploinsufficiency (and deficiency) of SAMD9L impaired this process, thus, it is strongly suggested that SAMD9L dysfunction sustains receptor-mediated intracellular signaling, leads to prolonged cell survival, and eventually develops MDS and related diseases. These findings lead us to the idea that a molecule that suppresses cytokine-mediated activation might be a therapeutic possibility for diseases with *Samd9L*haploinsufficiency.

Prior to the study shown above, we already analyzed biological function of *Miki*, another gene located in the microdeleted region [4]. *In vitro*and *in vivo*analyses demonstrated that MIKI plays essential roles in centrosome maturation and proper chromosomal segregation [4]. Since *Samd9L*and *Miki*are frequently simultaneously deleted in patients with −7/7q− [2], it would be likely that deficiency of *Samd9L*and *Miki*cooperatively contributes to MDS development. To address this possibility, we are planning to generate mice haploinsufficient for both *Samd9L*and *Miki*.

Hematopoietic malignancies with chromosomal deletions, such as −5/5q− and −7/7q− syndromes, show complex clinical features and would be resulted from cumulative dysfunction of multiple genes. Haploinsufficiency of multiple responsible genes located within the large chromosome deletion(s) transforms myeloid progenitors into MDS and related diseases [1, 5]. Regarding −7/7q− syndrome, in addition to *Samd9L*and *Miki*, previous studies propose different candidate genes whose reduced expression and/or deregulation would be responsible for disease initiation and progression, such as *EZH2*[6], *IRF5*[7], and *CUX1*[8]. Therefore, to achieve a comprehensive understanding of MDS and related diseases with −7/7q−, it is necessary to define and characterize genes whose mutational events cooperatively contribute to the pathogenesis of the diseases, which would help us to develop novel therapies and clinical managements.
